# The impact of water consumption on hydration and cognition among schoolchildren: Methods and results from a crossover trial in rural Mali

**DOI:** 10.1371/journal.pone.0210568

**Published:** 2019-01-17

**Authors:** Anna N. Chard, Victoria Trinies, Caroline J. Edmonds, Assitan Sogore, Matthew C. Freeman

**Affiliations:** 1 Department of Environmental Health, Emory University Rollins School of Public Health, Atlanta, Georgia, United States of America; 2 School of Psychology, University of East London, London, United Kingdom; 3 Monitoring, Evaluation, and Learning Section, Save the Children Mali, Bamako, Mali; University of Texas Medical Branch at Galveston, UNITED STATES

## Abstract

Adequate provision of safe water, basic sanitation, and hygiene (WASH) facilities and behavior change can reduce pupil absence and infectious disease. Increased drinking water quantity may also improve educational outcomes through the effect of hydration on attention, concentration, and short-term memory. A pilot study was conducted to adapt field measures of short-term cognitive performance and hydration, to evaluate levels of hydration, and to investigate the impact of providing supplementary drinking water on the cognitive performance of pupils attending water-scarce schools in rural Mali. Using a cross-over trial design, data were collected under normal school conditions (control condition) on one visit day; on the other, participants were given a bottle of water that was refilled throughout the day (water condition). Morning and afternoon hydration was assessed using specific gravity and urine color. Cognitive performance was evaluated using six paper-based tests. Three percent of pupils were dehydrated on the morning of each visit. The prevalence of dehydration increased in the afternoon, but was lower under the water condition. Although there was a trend indicating drinking water may improve cognitive test performance, as has been shown in studies in other settings, results were not statistically significant and were masked by a “practice effect.”

## Introduction

Health and educational benefits associated with improved water, sanitation, and hygiene (WASH) in schools include reduced diarrhea, absence, acute respiratory infection, and soil-transmitted helminth infection [1–5]. The availability of water during the school day is essential for supporting personal hygiene, sanitation, and maintaining a clean school environment. Increased access to water for drinking at school may also directly affect pupils’ academic performance through the cognitive benefits associated with decreased dehydration [6–8].

A recent UNICEF report found that only 53% of schools in least developed and other low-income countries had access to adequate water facilities, highlighting a gap in access to year-round, reliable, and safe water supply in sufficient quantities to support students’ needs [9]. Two studies assessing dehydration prevalence among school-age children living in hot, arid regions found that approximately two-thirds of children were in a state of moderate to severe dehydration [10, 11].

The impact of dehydration on cognitive performance is well studied among adults in experimental settings. Dehydration induced through exercise or heat stress has been associated with decreased short-term memory [6, 8], long-term memory [8, 12], arithmetic efficiency [6], visuospatial function [6], and attention [7]. Few studies have investigated the relationship between dehydration and cognition in children. Evidence from three intervention studies in the United Kingdom corroborate findings among adults, suggesting that drinking water was associated with better scores of attention [13, 14], short-term memory [14–16], and visual search [13]. However, these studies did not collect biometric measures of hydration status. Two additional studies conducted among children in Israel and Italy that assessed hydration status through urine osmolality found that dehydration was associated with decreased short-term memory [10, 16].

Linking drinking water availability directly to cognitive skills among children in water-scarce areas would have important public health and policy implications. A deeper understanding of the relationship between hydration and cognition could provide significant and novel evidence for the importance of improving water access in schools. Here, we aim to address the gaps in existing literature by assessing the relationship between water consumption, hydration, and cognition in a setting where children do not commonly have water access during the school day.

We assessed the prevalence of dehydration among children attending schools in Mali, West Africa, and examined the effect of drinking supplementary water during the school day on hydration status and on cognitive test scores. Our hypothesis was that the majority of students would be dehydrated and that the provision of supplementary water would be associated with improved hydration and improved cognition. Methods included the piloting and refining of cognition measurements that had not been previously used in sub-Saharan African field settings. In addition, to our knowledge we collected one of the first sets of data indicating biometric levels of dehydration and reporting on the cognitive effects of dehydration in sub-Saharan Africa or elsewhere in the global South, where access to water is the poorest.

## Materials and methods

### Setting

We conducted a pilot study to investigate the impact of providing supplementary drinking water on the cognitive performance of pupils in water-scarce schools in rural Mali. The purpose of this study was to 1) pilot measures of short-term cognitive performance, 2) pilot field measures of hydration, 3) pilot data collection procedures for potential inclusion in a larger trial, 4) evaluate levels of dehydration among primary school students in water-scarce settings, and 5) test the association between drinking water and hydration on various measures of cognitive performance.

Data collection took place between January 7–10 and March 4–7, 2013 at two rural primary schools within 20 km of Sikasso town, Mali. Data collection at the second school was delayed due to armed conflict within the country. The maximum high temperature for data collection was 29°C in January and 40°C in March.

### School eligibility, school selection, and participant selection

Schools were eligible for inclusion if they had no water point access within 0.5 kilometers, were within 1.5 hours drive from Sikasso town, and had at least 60 students in grades three through six. Two schools meeting eligibility requirements were purposively selected based on logistical considerations.

A total of 120 pupils in grades five (ages 9–13) and six (ages 10–16) were recruited. At each school, 30 pupils from each grade were randomly selected from school rosters using random number lists. In the event a pupil was absent or did not wish to participate, we continued to select pupils randomly from the class rosters until a sample size of 30 was reached for each grade.

### Study design

We employed a crossover trial design in which each pupil in the study served as his or her own control. A crossover design was selected over a randomized controlled trial design due to the logistical challenge of randomizing water distribution within classrooms. Given the novel study procedures, crowded school setting, and limited timeframe, we were not certain that we could ensure water was not shared between pupils in intervention and control groups.

Hydration and cognition measurements were collected on two different days at each school. On one of the visit days we collected data without changing any conditions at the school (the control condition). On the other visit day we provided all pupils, regardless of participation in the study, with a 1.5 litre bottle of water in the morning, encouraged them to drink throughout the day, and refilled their bottle upon request (the water condition). We did not track the amount of water each pupil consumed. To account for confounding due to becoming familiar with the test (henceforth referred to as “practice effect”), the order of intervention days was counterbalanced between schools so that one school received water on the first day, while the other received water on the second day. Additionally, we included a separation of three days between visits.

To evaluate potential confounders or effect modifiers of hydration and cognition, participants were asked if they had anything to eat or drink that morning and reported drinking water availability at school. Staff members also made observations of drinking water availability at the school on the day of the visit. The majority of pupils went home at noon and returned for afternoon classes. We did not record lunch practices.

### Measures of hydration

We collected three measures of hydration: urine specific gravity (U_sg_), urine color (U_col_), and self-reported thirst. Both U_sg_ and U_col_ are inexpensive measurements that can be easily conducted in the field with minimal training. They are strongly correlated with urine osmolality [17, 18], a common measure of hydration in non-laboratory settings [10, 11, 16]. U_sg_ measures urine density compared with water and was measured with ATAGO MASTER-URC/NM urine specific gravity analog refractometers (model 2793, ATAGO U.S.A. Inc., Bellevue, WA) [18]. The refractometers were calibrated using distilled water and were recalibrated at least every 15 readings, according to manufacturer instructions. U_col_ was measured against a validated scale of eight colors [17, 18]. Two trained enumerators independently evaluated each sample, and re-evaluated the sample together if their independent values differed; a third trained enumerator was consulted if no consensus was reached. Self-reported thirst [13, 19] was collected on a five-point pictorial scale based on the Wong-Baker FACES pain rating scale [20]. For analysis, the least-thirsty image was assigned a value of 5 and values decreased to 1 as reported thirst increased.

Pupils provided urine samples between 8 and 9 am and again between 2–3 pm on each day of data collection. All urine analyses were conducted on the school grounds by trained study enumerators. Pupils self-reported thirst in the afternoon, after the completion of cognitive testing.

### Measures of cognition

Cognition was measured using six tasks that assessed visual attention, visual memory, short-term memory, and visuomotor skills. These tests were taken from previous research on hydration and cognition that was conducted with children in Israel and the United Kingdom [10, 13, 14], piloted in Mali, and adapted to the Malian context.

#### Letter cancellation

This test assesses *visual attention*. Pupils were given a grid containing target letters randomly dispersed among non-target letters and were given one minute to cross out as many target letters as possible. Scores were calculated by subtracting the number of non-target letters identified from the number of target letters identified; the maximum test score was 38.

#### Direct image difference

This test assesses *visual attention*. Two nearly identical pictures were presented side-by-side. Pupils were given one minute to circle differences between the two images. Scores were calculated by subtracting the number of incorrect differences identified from the number of correct differences identified; the maximum test score was 9.

#### Indirect image difference

This test assesses *visual memory*. Two nearly identical pictures were presented in sequence. Pupils were given ten seconds to study the first image. They were then briefly presented with a blank page, followed by a second image, and given one minute to circle the differences between the two images on the second image, without returning to the first. Scores were calculated by subtracting the number of incorrect differences identified from the number of correct differences identified; the maximum test score was 9.

#### Forward digit recall

This test assesses *short-term memory*. Twelve sequences of numbers two to seven digits in length were read aloud to pupils at a rate of one number per second. Pupils were asked to write down the sequence in order after the sequence was read aloud. Two scores were derived from this test: the total number of correctly recalled sequences (maximum score of 12) and the maximum digit span of the correctly recalled sequence (maximum score of 7).

#### Reverse digit recall

This test assesses *short-term memory*. Ten sequences of numbers two to five digits in length were read aloud to pupils at a rate of one number per second. Pupils were asked to write down the sequence in reverse order after the sequence was read aloud. Two scores were derived from this test: the total number of correctly recalled sequences (maximum score of 10) and the maximum digit span of the correctly recalled sequences (maximum score of 5).

#### Line tracing task

This test assesses *visuomotor skills*. Pupils were presented with two curved parallel lines. They were given fifteen seconds to draw a line between them as quickly as possible while attempting not to touch the printed lines. Scores were calculated by subtracting the number of times the pupil’s line touched the side from the total length of the line in centimeters; the maximum test score was 29.

All cognitive tests were paper-based and administered by trained study staff in a group setting within the school classrooms. Testing sessions were standardized using written scripts. Staff introduced each test with a scripted explanation and an example, with no breaks between tests. Testing sessions lasted a total of 60–75 minutes and began at 3:00 pm in the afternoon of each visit. Each pupil in the study completed the testing session twice, once on the control condition day and once on the supplementary water condition day. Four parallel versions of each test were developed so that individual pupils did not receive the same test twice and pupils sitting next to each other did not receive the same test. All four test versions were distributed at each testing session. Tests were independently graded by two different staff members using fixed criteria. Grading criteria also provided guidelines to indicate whether or not pupils understood the tasks according to instruction. Tests with conflicting scores were examined by the study coordinator, who decided the final score for the task.

### Data analysis

Data were entered into MS Excel and analyzed using STATA 13 SE. We tested both the impact of treatment condition (whether student was provided water or not during the day) and hydration status on change in test score. U_sg_ was used to test the impact of hydration on change in test score because it was the only of our three hydration measures based on biomarkers, and is the most accurate of those three measures of hydration status [21]. A higher U_sg_ indicates increased dehydration. Pupils were classified as dehydrated if they had a U_sg_ of 1.020 or higher, which is equal to the dehydration threshold of urine osmology>800 mOsmol kg-1 H_2_O that has been used in previous studies of dehydration among children [10, 11, 16]. A total of eight scores for the six cognitive tests were calculated according to grading criteria. Scores were coded such that higher test scores on all cognitive tests represented better performance.

### Univariable analysis

As proof of concept of the effect of water provision on hydration, we evaluated univariable differences in morning and afternoon hydration, U_sg_, and U_col_ by treatment group using McNemar’s test statistic (binary variables) and paired sample t-tests (continuous variables). To evaluate the correlation between U_sg_, U_col_, and self-reported thirst, as well as the correlation between each of the cognitive test scores, pairwise tests of correlations between cognitive test scores were conducted using the pwcorr command. Lastly, to measure the presence of a “practice effect,” paired sample t-tests were used to assess differences in cognitive test scores between school visits.

### Multivariable analysis

We examined the association between the provision of supplementary drinking water (treatment) and cognitive test scores as well as the association between pupil hydration (regardless of treatment) and cognitive test scores. These associations were assessed using separate mixed-effects linear regression models, where each cognitive test was the outcome, while treatment condition or hydration status, respectively, was the predictor covariate. Models included a random intercept at the pupil level to account for pupils acting as their own control. Unstandardized Beta coefficients are presented.

All models adjusted for multiple comparisons using the Bonferroni correction; as such, associations were considered significant if they had a *p*-value <0.006, the alpha necessary to reach 95% significance with eight hypotheses. Models were assessed for interaction and confounding with the following variables chosen *a priori*: pupil sex, pupil grade, reported drinking in the morning, reported eating in the morning, reported thirst, and morning hydration.

Interaction was assessed by running models of each cognitive test outcome with each predictor variable, potential interaction covariate, and an interaction term for the predictor and covariate (e.g. treatment*sex). Some variables initially indicated interaction at *p*<0.05. However, after adjusting for multiple comparisons using the Bonferroni correction, the only effect modifier to retain significance was pupil sex, which modified the relationship between afternoon dehydration and forward number recall- maximum digit span test score. Stratified results from this model are presented. All other associations were then tested for confounding; covariates significantly associated with the predictor variable as well as the outcome variable in independently run fixed-effect models were considered to be confounding variables. At *p* = 0.006, grade confounded the association between treatment and direct image difference & indirect image difference test scores, so was included as a control variable in these models. All models controlled for the visit day in order to account for a “practice effect” on cognitive tests.

We compared models from all pupils to models that excluded scores from pupils who did not complete cognitive tests according to instruction. There were no significant differences between model results, thus, we present the former results in order to maximize sample size. Only students with complete data for all measures of interest were included in analysis. We dropped 13 pupils due to absence on the second day of data collection, not being able to provide a urine sample, or inability to match pupils test scores and hydration measures due to improper identification procedures.

### Ethics

This study was approved by Emory University’s Institutional Review Board (IRB00062354), the Mali Ministry of Education, and the National Technical and Scientific Research Center (*Centre National de la Recherche Scientifique et Technique*) in Mali (001/2013-MESRS/CNRST). All three institutions approved consent *in loco parentis* (in the place of parents) due to the logistical challenges of finding and contacting parents in their homes, risk of lost wages to parents if they were summoned to school, and low levels of literacy making letters unfeasible. Permission for study activities and approval of a waiver of parental consent was also obtained from the *Centres d’Animation Pédagogique* (Center for Pedagogical Activity) and *Académie d’Enseignement* (Academy of Education) in Sikasso, both local government representatives responsible for education in the area where the study was conducted. Prior to commencing study activities at each school, we obtained consent in *loco parentis* from the school director and the *Comité de Gestion Scolaire* (school management committee), the organization empowered to oversee management and activities at the school, on behalf of the community that school serves. Pupils who were selected for the study provided informed verbal assent in a private setting prior to the start of data collection activities.

## Results

### Study population

Data were collected from 120 pupils in two schools; of these, 107 (89.2%) pupils had complete data and were included in analysis. The sample was initially comparable in terms of sex, grade, and school. After removing pupils with incomplete data (n = 13), the final sample included 46 (43.0%) girls, 61 boys (57.0%); 58 (54.2%) pupils from grade five, 49 (45.8%) pupils from grade six; 47 (43.9%) from School 1, and 60 (56.1%) from School 2. The mean (sd) age was 11.6 (1.0) years in School 1 and 12.1 (1.7) years in School 2.

### Univariable estimates of association with hydration

Only 3% of pupils were classified as dehydrated in the morning according to U_sg_ (U_sg_>1.019), regardless of visit day or study condition. The difference between water and control condition mean morning U_sg_ or U_col_ was not statistically significant, and we found no difference in the prevalence of dehydration prior to distribution of water.

Pupils became more dehydrated throughout the school day under both study conditions. There was no significant difference in U_col_, self-reported thirst, or the prevalence of pupils classified as dehydrated in the afternoon under the water condition compared to the control condition. However, mean afternoon U_sg_ was significantly higher under the control condition compared to the water condition (Table 1). U_sg_ and U_col_ were strongly correlated both in the morning (r = 0.777, *p*<0.001) and afternoon (r = 0.734, *p*<0.001). Self-reported thirst, which was only measured in the afternoon, was not significantly correlated with either afternoon U_sg_ (r = 0.089, *p* = 0.20) or afternoon U_col_ (r = -0.003, *p* = 0.97).

**Table 1:** Univariable associations between hydration indicators and study conditon (n = 107).

	Water	Control	p*
Mean (SD) morning urine specific gravity (U_sg_)	1.008 (0.01)	1.007 (0.01)	0.35
Mean (SD) morning U_col_ (scale 1–7)	2.34 (1.54)	2.29 (1.24)	0.77
Dehydrated^†^ in morning	4 (3.7%)	2 (1.9%)	0.69
Mean (SD) afternoon U_sg_	***1*.*010 (0*.*01)***	***1*.*014 (0*.*01)***	***<0*.*01***
Mean (SD) afternoon U_col_ (scale 1–7)	3.10 (1.82)	3.44 (1.43)	0.11
Dehydrated^†^ in afternoon	12 (11.2%)	17 (15.9%)	0.38
Mean (SD) afternoon self-reported thirst (scale 1–5)	3.2 (1.5)	3.1 (1.6)	0.21

*p-value based on McNemar’s test statistic for binary variables and paired sample t-tests for continuous variables

^†^Pupils with a U_sg_ >1.019 classified as mildly dehydrated

***Bold*** values indicate a significant association at α = 0.05

### Univariable estimates of association with cognition

Results from pairwise tests of correlations between cognitive test scores and results from the paired t-tests of the association between test score and visit day are shown in Table 2. Most tasks were significantly correlated with at least one other task included in the battery of cognitive tests. Students achieved significantly higher scores on the second visit compared to the first visit for six of the eight cognitive tests, regardless of treatment condition.

**Table 2:** Correlation matrix of cognitive test scores’ pairwise correlation coefficients and univariable associations between visit day and mean (standard devation) of cognitive test scores.

	Pairwise Correlation Coefficents								Mean (SD) test scores		
	LC^1^	DID^1^	IID^2^	NFC^3^	NFM^3^	NRC^3^	NRM^3^	LT^4^	Visit 1	Visit 2	p*
Letter cancellation (LC)^**1**^	1								***18*.*9 (7*.*6)***	***26*.*3 (7*.*0)***	***<0*.*01***
Direct image difference (DID)^**1**^	0.1890	1							***1*.*4 (1*.*4)***	***2*.*3 (1*.*6)***	***<0*.*01***
Indirect image difference (IID)^2^	0.1668	***0*.*3137***	1						***1*.*6 (1*.*5)***	***2*.*3 (1*.*6)***	***<0*.*01***
Forward number recall- total (NFC)^3^	0.1465	0.1775	***0*.*2985***	1					***5*.*0 (1*.*4)***	***5*.*5 (1*.*5)***	***<0*.*01***
Forward number recall- maximum digit span (NFM)^3^	0.1360	***0*.*2228***	***0*.*2975***	***0*.*7235***	1				***4*.*1 (1*.*0)***	***4*.*3 (1*.*0)***	***0*.*04***
Reverse number recall- total (NRC)^3^	0.0870	0.0885	0.2052	***0*.*3310***	***0*.*2473***	1			3.4 (1.8)	3.6 (1.6)	0.64
Reverse number recall-maximum digit span (NRM)^3^	0.1013	0.0234	***0*.*2128***	***0*.*2208***	0.1705	***0*.*8440***	1		3.2 (1.3)	3.3 (1.1)	0.41
Line trace (LT)^4^	***0*.*2302***	0.1049	0.0947	0.0704	0.0961	-0.0693	-0.0858	1	***13*.*4 (7*.*8)***	***17*.*2 (6*.*0)***	***<0*.*01***

***Bold values*** indicate a significant association at α = 0.05

*p-value based on paired t-tests

Target skills assed by test:

^1^visual attention

^2^visual memory

^3^short-term memory

^4^visuomotor skills

### Multivariable estimates of association between cognitive test scores and treatment condition

In adjusted models, the provision of supplementary drinking water was significantly associated with two cognitive tests: reverse number recall (total) and line trace. Under the water condition, pupils performed better on the reverse number recall test. However, pupils had lower scores on the line trace test under the water condition (Table 3).

**Table 3:** Mixed effects linear regression models of associations between treatment group, afternoon measures of hydration, and cognitive performance (n = 107).

Cognitive Test	Treatment			Dehydrated*			Urine Specific Gravity (U_SG_)		
	Beta	95% CI	p	Beta	95% CI	p	Beta	95% CI	p
Letter cancellation	0.36	-0.81, 1.53	0.545	-1.95	(-4.23, 0.32)	0.092	-64.98	(-177.47, 47.51)	0.258
Visit (ref: Visit 1)	***7*.*43***	***6*.*26*, *8*.*59***	***<0*.*001***	***7*.*40***	***(6*.*26*, *8*.*54)***	***<0*.*001***	***7*.*28***	***(6*.*12*, *8*.*44)***	***<0*.*001***
Image difference, direct	0.25	-0.10, 0.60	0.163	-0.47	(-1.05, 0.10)	0.103	-18.22	(-46.28, 9.84)	0.203
Visit (ref: Visit 1)	***0*.*90***	***0*.*55*, *1*.*25***	***<0*.*001***	***0*.*87***	***(0*.*52*, *1*.*22)***	***<0*.*001***	***0*.*84***	***(0*.*49*, *1*.*19)***	***<0*.*001***
Pupil grade (ref: 5^th^ grade)	***0*.*77***	***0*.*36*, *1*.*19***	***<0*.*001***	—	—	—	—	—	—
Image difference, indirect	0.26	-0.09, 0.61	0.151	0.30	(-0.29, 0.89)	0.323	6.81	(-22.33, 35.95)	0.647
Visit (ref: Visit 1)	***0*.*74***	***0*.*39*, *1*.*09***	***<0*.*001***	***0*.*71***	***(0*.*36*, *1*.*06)***	***<0*.*001***	***0*.*72***	***(0*.*37*, *1*.*08)***	***<0*.*001***
Pupil grade (ref: 5^th^ grade)	***0*.*70***	***0*.*25*, *1*.*15***	***0*.*002***	—	—	—	—	—	—
Number recall total, forward	-0.01	-0.32, 0.30	0.943	0.22	(-0.32, 0.77)	0.420	17.35	(-9.31, 44.02)	0.202
Visit (ref: Visit 1)	***0*.*52***	***0*.*21*, *0*.*83***	***0*.*001***	***0*.*52***	***(0*.*21*, *0*.*83)***	***0*.*001***	***0*.*55***	***(0*.*24*, *0*.*86)***	***0*.*001***
Number recall maximum digit span, forward	0.01	-0.20, 0.22	0.936	0.17	(-0.20, 0.55)	0.359	4.61	(-13.70, 22.93)	0.621
Visit (ref: Visit 1)	***0*.*23***	***0*.*01*, *0*.*44***	***0*.*038***	0.22	(0.01, 0.44)	0.040	0.23	(0.02, 0.45)	0.034
Number recall total, reverse	***0*.*61***	***0*.*19*, *1*.*03***	***0*.*005***	0.19	(-0.48, 0.87)	0.577	-8.29	(-41.39, 24.80)	0.623
Visit (ref: Visit 1)	0.18	-0.25, 0.60	0.412	0.10	(-0.33, 0.53)	0.648	0.09	(0.35, 0.53)	0.687
Number recall maximum digit span, reverse	0.17	-0.15, 0.48	0.296	0.08	(-0.39, 0.54)	0.746	3.12	(-19.64, 25.89)	0.788
Visit (ref: Visit 1)	0.15	-0.16, 0.47	0.346	0.13	(-0.18, 0.44)	0.415	0.14	(0.18, 0.45)	0.399
Line trace	***-4*.*48***	***-5*.*87*, *-3*.*08***	***<0*.*001***	1.29	(-1.38, 3.96)	0.344	79.16	(-53.10, 211.41)	0.241
Visit (ref: Visit 1)	***-4*.*48***	***1*.*93*, *4*.*73***	***<0*.*001***	***3*.*81***	***(2*.*20*, *5*.*43)***	***<0*.*001***	***3*.*96***	***(2*.*34*, *5*.*58)***	***<0*.*001***

*Pupils with a U_SG_ >1.019 classified as dehydrated

***Bold*** values indicate a significant association at α = 0.006, the level of 95% significance after correcting for multiple comparisons

Models include a random intercept at the pupil level to account for clustering

### Multivariable estimates of association between cognitive tests scores and hydration status

We examined the impact of hydration on cognitive test performance, regardless of treatment condition. Neither hydration status, where a U_sg_ greater than 1.019 indicated dehydration, nor U_sg_ were significantly associated with any cognitive test score (Table 3). The test for interaction indicated that pupil sex significantly modified the association between forward number recall (maximum) and afternoon dehydration. When stratified by sex, males performed worse when dehydrated (β = -0.14; 95% CI -0.54, 0.27; *p* = 0.501) and females performed better when dehydrated (β = 1.10; 95% CI 0.31, 1.89; *p* = 0.006); only the association between hydration and forward number recall among female pupils approached statistical significance.

## Discussion

We conducted a cross-over trial as part of a pilot study to examine the associations between water consumption, hydration, and cognition among pupils attending water-scarce schools. We successfully adapted measures of cognitive performance that could be completed by children in rural Malian schools and tested the feasibility of field hydration measures and data collection procedures within schools in Sub-Saharan Africa. Results demonstrated that supplementary water provision within a school setting significantly decreased U_sg_, even within a short time period. However, we found no effect of the impact of supplementary water provision on cognitive test scores.

This research refined a battery of cognitive tests for use with children in Mali which can be adapted to other developing settings. Research conducted in the U.K. concluded that their cognitive test of visual memory was too easy for the target population, indicated by many children achieving the maximum score on the test, and thus modifying study results [13]. Our results show that the percentage of children achieving the maximum score or the minimum score on any of the cognitive tests ranged from 0.5%-15.4% and 0.5–4.2%, respectively, indicating that the cognitive tests adapted for this trial were neither too difficult nor too hard. However, results from our pairwise tests of correlation indicate that the two tests measuring visual attention (letter cancellation and direct image difference) were not significantly correlated, suggesting that further adaptation may be needed on these tests to measure this target skill. Furthermore, while scores for each of the four tests measuring short-term memory were significantly associated with at least one other score in the suite of tests measuring that domain, they were very similar tests in that they all incorporated number recalls. Thus, correlation does not necessarily indicate that they were in fact measuring the cognitive skill they were intended to measure.

This is one of the first studies to employ existing field methodology to collect urine samples and measure dehydration among school children in low-resource school settings. Results from this pilot study were further refined in a subsequent trial in Zambia [22]. Prior research on dehydration among schoolchildren has relied predominantly on self-reported thirst as their measure for dehydration. Although evidence- particularly among healthy individuals- is limited, research has concluded that one’s thirst response is not an accurate measure of hydration [23, 24]. We found no research investigating this association among children. Our results demonstrated no significant difference between self-reported thirst among pupils under the water condition compared to the control condition, even though the measurements of U_sg_ indicated that pupils under the water condition had significantly higher levels of hydration than pupils under the control condition. Additionally, self-reported thirst and the biometric measurement of U_sg_ were not significantly correlated. These findings support previous literature concluding that self-reported thirst is not an accurate measure of hydration. Given our findings, future research should consider utilizing only measurements that provide biometric evidence of dehydration. Data also revealed that U_col_, although strongly correlated with U_sg_, did not capture a significant difference in afternoon hydration between water and control conditions. We believe this may have been due to the subjective nature of matching urine color to the color chart. The use of refractometers to measure U_sg_ required less training and took less time than measuring U_col_, and thus is recommended for future studies investigating dehydration levels of subjects in low-resource settings.

Our finding that only 2.8% of pupils were dehydrated in the morning stands in stark contrast to previous research which reported that 84% of Italian school children [16], 68% of Israeli school children [11], and 43% of Zambian schoolchildren [22] were dehydrated at the beginning of the school day. While this result was initially surprising, it may be partly explained by evolutionary mechanisms. In their research, Bar-David reported that among their sample of Israeli schoolchildren, Bedouin children, who originate from a population that has lived in the desert for many generations, had the lowest mean urine osmolality (the lowest prevalence of dehydration), possibly because their bodies adapted over time to have a lower threshold of thirst [10, 11]. Thus, Malian children, who reside in hot, arid, and water-scarce environments, may have also adapted a greater resistance to dehydration, leading to a lower prevalence of dehydration at the beginning of the school day. Extremely low levels of morning dehydration may also be partly explained by the fact that a vast majority of students (93%) reported drinking something in the morning before going to school. We do not believe that pupils intentionally consumed more water than usual in preparation for participation in the research. Neither school officials nor pupils were aware of the study topic, activities, or pupil selection prior to the first day of the study. Thus, participants would not have had the foreknowledge to alter their normal drinking behaviors. Although school officials and pupils were aware of the date of the second visit, given that no significant differences in the prevalence of dehydration or U_sg_ were observed between the first and second visits, it is unlikely that students changed their drinking practices for the second day.

Under both treatment conditions, dehydration increased throughout the day. Pupils had significantly lower U_sg_ in the afternoon under the supplementary water condition than under the control condition, demonstrating the “proof of principle” that supplementary water provision improves hydration. However, there was no significant difference in the prevalence of afternoon dehydration among pupils in the water group compared to pupils in the control group. Nonetheless, when the significant impact of water consumption on increasing U_sg_ is considered in light of findings of the relationship between drinking water and cognition from other contexts [13–16], there is evidence that providing drinking water at school may create a positive impact on pupil learning.

We found some evidence that supplementary water provision was associated with higher scores on cognitive tests, but few results were significant. These results are consistient with those from our follow-up trial among primary school children in Zambia [22]. Treatment was significantly associated with higher scores on the letter cancellation task, a result supported by previous literature that also found a positive relationship between provision of drinking water and performance on visual attention tasks [14, 22]. While previous studies have reported no significant association between water provision and visuomotor skills [13, 14], we found that scores on the line trace test were significantly, but negatively associated with supplementary water provision. Although this result was unexpected, it may be largely explained by a practice effect, in which pupils performed significantly better the second time they took the test, regardless of treatment condition. Although pupils took a different version of the test on each day, a practice effect was evident, as test scores significantly improved when pupils performed each task the second time. One possible reason for this difference could be that pupils in Mali are not accustomed to the types of activities performed during the tests, which were adapted from tests used in Western settings. Although the distribution of test scores and the correlation of tests measuring the same domain do indicate that the tests were suitably adapted to the context, the novelty of the tests may have caused a much lower baseline score at the first testing session. Pupils may need to practice completing the tasks several times in order to fully understand the tests before their scores are measured.

Lastly, evidence on the degree and duration of dehydration necessary to impact cognitive performance is limited. It is possible that the lack of significant improvements in cognitive performance following treatment is because one school day of supplementary water provision is not sufficient to reverse the impacts of chronic dehydration and impart cognitive benefits on schoolchildren; perhaps more long term water consumption is necessary for these benefits to be measurably improved [22]. Further, although the U_sg_ data provide evidence that pupils drank under the treatment condition, we did not measure the volume of water consumed by subjects. Measuring the volume of water consumed by subjects and including a dose-response measure in the analysis could contribute to the discourse on how much water consumption is needed to improve hydration, and how much hydration is needed to improve cognition.

### Limitations

There are several limitations to the current research. First and most crucial was the impact of the practice effect, in which pupils performed significantly better on cognitive testing during the second visit, regardless of treatment condition. Approaches to limit or account for the practice effect on cognitive testing in primary school populations residing in settings where this type of testing is uncommon requires additional attention; future research should focus on alternative trial designs to minimize this impact. Additionally, the fixed test order could have led to a learning effect across tests, where certain tests- conceivably later on in the series- revealed a more significant association due students becoming more comfortable with testing in general, rather than due to the skill tested. Students in both the intervention and control would have had the same learning effect, which would bias our results to the null, but there is no way to control for this within the individual models. However, we observed no trend where students performed differently on tests administered in the end of the suite on either testing day. Further, we reviewed the estimates of effect and do not find any effect modification. Second, because this was a pilot study, the sample was limited to 120 pupils in two schools. As such, the study may not have been sufficiently powered to detect significant but less strong impacts of supplementary water provision or hydration status on cognitive performance. Low levels of dehydration across study groups may have also further limited our ability to detect an impact. Third, we conducted an intention-to-treat analysis and did not measure or control for the volume of water consumed by the participants in the treatment group. We did not measure whether pupils in the control group consumed water brought from home, and we could not ethically restrict them from drinking water. We also did not record lunch practices among students, and cannot guarantee that children did not consume water when they went home for lunch. As such, we cannot unequivocally state that the intervention and control groups were separated by water consumption, or lack thereof. However, afternoon U_sg_ was collected regardless of treatment condition, and results validate the degree of water consumption under treatment. Additionally, lunch practices among individual students would likely be similar across days, thus the influence of lunch practices would be consistent across test conditions since pupils act as their own controls. Fourth, due to external events, data collection at the second school was delayed for two months and occurred during a warmer period. The higher temperatures during the second data collection period may have impacted study results. Evidence suggests that exposure to heat may independently impact cognitive functions, however this research has not been conducted among children [25–27]. Although significantly more pupils in the second school were dehydrated in the afternoon compared to pupils in the first school, due to the crossover design, it is not possible to quantify the effect that temperature may have had on study outcomes. Last, the methodology, including the duration of tests, were adapted from cognitive tests previously used among primary school children [13, 14, 28], but the total testing time was longer than in previous studies due to the novelty of the tests in the population and our emphasis on explanation and examples. However, because there was no significant trend in scores across the testing suite, there is no evidence that performance worsened due to fatigue among students.

We suggest a two-step approach for collecting further evidence on hydration and cognition among pupils in water-scare schools. First, we recommend implementing a second trial with cognitive testing methodology that addresses the challenges of the practice effect in order to increase the evidence base on the link between hydration and cognition among schoolchildren in water scare areas. Once the link between improved hydration and cognition among schoolchildren has been established under experimental conditions, we recommend carrying out cross-sectional hydration testing in a larger sample of schools. Considering the apparent invalidity of self-reported thirst and the subjective nature of urine color evaluation, we recommend the use of urine specific gravity or another objective biometric measure for hydration testing. Given the evidence previously established, hydration in this case would serve as an easily quantified and measured proxy for pupil attention, memory, and concentration. Findings from this investigation could provide evidence of the benefit of drinking water access, and specifically on the construction of water points on school grounds, for pupils’ educational attainment.

## Conclusions

This study represents novel research across multiple scientific disciplines and development sectors, and is an important step in developing clear and direct linkages between provision of WASH in schools and learning. Results demonstrated the proof of principle that increased water access improves hydration. Although we found no evidence for our hypothesis that improvements in hydration status leads to improvements in cognitive performance among pupils in water scare schools, results may have been masked by a strong practice effect, and the power to detect significant differences was limited. We demonstrated the feasibility of collecting biometric measurements of hydration status and testing cognitive abilities in resource-poor settings. Findings from this research and subsequent studies of hydration and cognition have broad significance for advocacy for international development and health sectors for increased attention to insufficient access to water supply for school children.

## Supporting information

S1 File Data. (XLSX)
